# Deep Layered Network Based on Rotation Operation and Residual Transform for Building Segmentation from Remote Sensing Images

**DOI:** 10.3390/s25082608

**Published:** 2025-04-20

**Authors:** Shuzhe Zhang, Taoyi Chen, Fei Su, Hao Xu, Yan Li, Yaohui Liu

**Affiliations:** 1 School of Surveying and Geo-Informatics, Shandong Jianzhu University, Jinan 250101, China; 2023165101@stu.sdjzu.edu.cn (S.Z.); sufei21@sdjzu.edu.cn (F.S.); liuyaohui20@sdjzu.edu.cn (Y.L.); 2 The 54th Research Institute of CETC, 589 Zhongshan West Road, Shijiazhuang 050081, China; 3 Shandong Academy of Agricultural Sciences, Jinan 250100, China; xuhao@saas.ac.cn; 4 National Engineering and Technology Center for Information Agriculture, Nanjing Agricultural University, Nanjing 210095, China; 2023201117@stu.njau.edu.cn

**Keywords:** building segmentation, transformer, remote sensing image, attention mechanism, deep layer enhanced

## Abstract

Deep learning has been widely applied in building segmentation from high-resolution remote sensing (HRS) images. However, HRS images suffer from insufficient complementary representation of target points in terms of capturing details and global information. To this end, we propose a novel building segmentation model for HRS images, termed C_ASegformer. Specifically, we design a Deep Layered Enhanced Fusion (DLEF) module to integrate hierarchical information from different receptive fields, thereby enhancing the feature representation capability of HRS information from global to detailed levels. Additionally, we introduce a Triplet Attention (TA) Module, which establishes dependency relationships between buildings and the environment through multi-directional rotation operations and residual transformations. Furthermore, we propose a Multi-Level Dilated Connection (MDC) Module to efficiently capture contextual relationships across different scales at a low computational cost. We conduct comparative experiments with several state-of-the-art models on three datasets, including the Massachusetts dataset, the INRIA dataset, and the WHU dataset. On the Massachusetts dataset, C_ASegformer achieves 95.42%, 85.69%, and 75.46% for OA, F1score, and mIoU, respectively. C_ASegformer shows more accurate performance, demonstrating the validity and sophistication of the model.

## 1. Introduction

Building segmentation aims to distinguish building footprints from high-resolution remote sensing (HRS) images. Due to its potential applications, building segmentation has also been extended to urban planning, earthquake prevention and mitigation, and environmental monitoring [1]. It can provide an important reference for urban planning decisions, environmental monitoring, disaster early warning, etc. [2,3]. The labor cost associated with building segmentation tasks, however, is relatively high. With the increasing prevalence of deep learning techniques, employing deep learning methods to accomplish building segmentation tasks has gradually become the prevailing trend [4].

In recent years, deep learning methods have made significant breakthroughs in tasks such as semantic segmentation [5] and deep convolutional neural networks (CNNs), and full convolutional neural networks (FCNs) have demonstrated considerable performances [6], with better results in the direction of building segmentation [7]. However, due to stacked convolutional layers, these methods lose detail information and reduce downsampling accuracy [8,9,10]. With the advancement of deep learning models, a feature extraction architecture with reference to FCN was proposed by He et al. [11]. The architecture employs residual concatenation, Atreus convolution and pyramidal scene parsing pooling strategies to increase semantic information. Later proposed SegNet [12], DeconvNet [13], U-Net [14], DeepLabV3+ [15], HRNet [16], MA-FCN [17] and MANet [18] were inspired by FCN. Some researchers further applied these methods to the automatic extraction of buildings in HRS images. Huang et al. added up-sampling and dense connectivity operations to the inverse convolutional layer to enhance the connectivity of the layers at different scales to obtain building segmentation results [19]. Zheng et al. demonstrated that foreground modeling is also very helpful for small object detection. The main idea in this regard is to simultaneously improve multi-scale feature representation and feature fusion capabilities [20]. Xu et al. used bootstrap filtering to filter feature impurities to further optimize the building segmentation results [21]. Although the above methods have achieved a lot in segmenting buildings from HRS images, applying them to segment buildings from HRS images still suffers from some problems of incomplete feature details. Different tones and textures of the same building in the image can lead to incomplete or partially missing extracted buildings [5]. To address the impact of complex targets on extraction accuracy, Tang et al. proposed a method that considers the bidirectional dependency between semantics and changes [22]. The model integrates a semantic-enhanced contrastive learning module and a dual-temporal semantic association capture mechanism, effectively identifying target information in complex environments. Beyond environmental factors, the shape of buildings themselves also increases the difficulty of model extraction. Wei et al. [23] introduced an end-to-end approach that utilizes feature lines as geometric primitives. By restoring the topological relationships among these lines within individual buildings, the method achieves the extraction of polygonal buildings. Du et al. [24] proposed a spatial cognitive graph convolution model, which vectorizes buildings to obtain location coordinates, enriching topological embeddings and enhancing the model’s generalization capability. Li et al. [25] introduced a novel uncertainty-aware network (UANet) to address the challenge of uncertain predictions that the model faces, which fails to accurately distinguish the complete footprints of buildings from complex geographical distributions, leading to significant omissions. UANet first determines the uncertainty levels of the foreground and background through a prior information guidance module and an uncertainty grading algorithm, then refines features to obtain segmentation maps by combining them with an uncertainty-aware fusion module. The limitations of CNN models lie in their excessive parameter count and inadequate ability to effectively capture global information.

With the rapid development of convolutional neural networks, both the structure of the model and the type of convolution is also being improved. In addition to the normal 2D convolution, Deconvolution Convolution [26], Atrous Convolution [27], and Depthwise Separable Convolutions (DSCs) [28] are proposed. Deconvolution Convolution is the inverse process of the convolution operation, which restores low-resolution feature maps to their original size. Atrous Convolution is a special type of convolution that extends the receptive field of a filter by adding fixed-interval zero-valued points to the interior of the filter [29], which increases the size of the receptive field that extracts more contextual information. Atrous Convolution effectively improves the size of the feature map and reduces the number of parameters, resulting in a larger sense field and stronger representation. DSC is a technique that effectively reduces the number of parameters in the convolution computation by decomposing the standard convolution into two steps: Depthwise Convolution and Point-by-point convolution [30]. Depthwise convolution reduces the parameter count by conducting convolution operations on individual input channels, while Point-by-point Convolution decreases channel numbers through the use of a 1 × 1 convolution kernel. DSC can enhance feature extraction capability effectively, while maintaining low computational and memory demands. This makes it crucial in resource-constrained scenarios, such as mobile devices, where efficient utilization of resources is essential.

HRS images have complex topography and the phenomenon of occlusion and overlapping of target features [31], so the convolutional neural network cannot accurately judge complex environments, while the attentional mechanisms have a similar ability to the ability of a human being to focus. It can extract buildings more accurately in complex environments. With the development of the attention mechanism, researchers have developed many plug-and-play attention modules, such as SENet [32], ECANet [33], CBAM [34], and so on. Furthermore, extracting multi-scale information for fusion also solves the above problem. Hebert et al. proposed a typical plug-and-play module called ASPP [35], using convolutional kernels with different sampling rat to capture multiscale information. Zhao et al. proposed the CBAM_ASPP module by fusing the ASPP module with the attention module CBAM [36]. It fuses feature information of different scales with weights, which can better retain important information features. In HRS images, the presence of abundant detailed information inevitably introduces irrelevant contextual data, which hinders the accurate inference of patch semantics. Li et al. [37] proposed a multi-head attention-attended module (AAM) that filters out irrelevant context by considering the correlation between self-attention maps and query vectors while simultaneously highlighting context that is rich in information. The attention mechanism effectively enhances the utilization of information extracted by the CNN backbone; however, it fails to address its limitation in capturing global information attention.

Recently, Transformer [38], originally designed for Natural Language Processing (NLP) tasks, has become a hot topic in the field of computer vision, i.e., Vision Transformer (ViT) [39]. Unlike the CNN structure, ViT converts a 2D image-based task into a 1D sequence-based task. Due to the powerful sequence-to-sequence modeling capability, ViT exhibits superiority over attention-based CNNs in extracting global contexts [40]. Xie et al. proposed a semantic segmentation model Segformer with ViT as backbone [41], constructed a lightweight decoder, and released MLP for feature aggregation [42]. Segformer emphasizes the robustness and effectiveness of the Transformer family of networks. Efficient and high-precision semantic segmentation, while facing the challenge of image interference, still remains challenging and have room to be improved [43]. He et al. [44] constructed pixel-level associations by setting the Transformer to a U-shape. Wu et al. [45] proposed a semantic segmentation model for HRS images that integrates Transformers with CNNs to achieve improved modeling of long-range spatial dependencies. Change et al. [46] designed a two-branch transformer to extract hierarchical and distinguishable unimodal features. The aforementioned approaches offer novel insights for HRS image tasks; however, there is still room for improvement in harnessing the available information.

In this paper, we propose the C_ASegformer model for building segmentation in HRS images, which employs a Transformer-based backbone for feature extraction. We introduce a Deep Layered Enhanced Fusion (DLEF) module to effectively integrate multi-scale information, thereby enhancing the model’s ability to capture complementary information across multiple levels. Furthermore, the Triplet Attention (TA) Module and the Multi-Level Dilated Connection (MDC) module establish multi-directional dependency relationships and contextual connections. To better evaluate the performance of the model, we conduct experiments on the Massachusetts buildings dataset [47], INRIA Dataset [48] and the WHU Building Dataset [49]. The C_ASegformer model is compared to mainstream models in the experiments.

The main contributions of this article are given as follows.

(1) The proposed DLEF module captures and integrates information from different receptive fields, effectively linking global and detailed information to achieve refined global representation.
(2) The TA module is introduced which establishes multi-directional dependency relationships through dimension rotation and residual transformations.
(3) The MDC module is proposed which constructs a bridge between multi-level information, enhancing information exchange and strengthening contextual relationships.

## 2. Methodology

### 2.1. Overview

**Figure 1:**
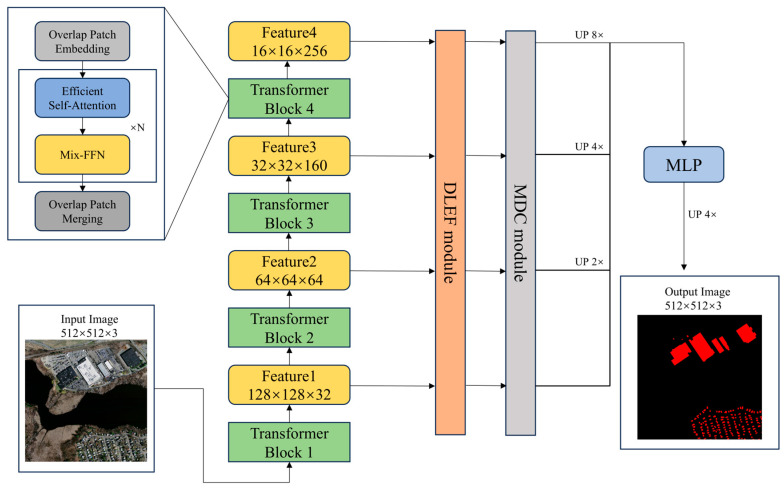
General framework of C_ASegformer.

In this study, we propose a semantic segmentation model, named C_ASegformer, which builds upon the Segformer model, utilizing the VIT to extract features. We present several enhancements to the C_ASegformer. First, we propose the DLEF module. This refined module not only captures multi-scale information within an image but also considers the interplay between multi-scale information through the attention mechanism, thus enabling improved extraction of semantic information from images. Second, we propose the MDC to address the disparity between low- and high-level semantic information and location information. The addition of the MDC further enhances the model’s performance. Finally, DSC is introduced to mitigate the issue of overfitting on small datasets by reducing the number of trainable parameters. The network structure of C_ASegformer is depicted in Figure 1. In order to better adapt to the task of building extraction in HRS images, considering both accuracy and training cost, we ultimately select MiT-B0 as the backbone of our model. The specific parameters and other main components are detailed in Table 1.

### 2.2. Segformer Structure

Segformer is a deep learning model architecture designed for image segmentation, featuring improvements and optimizations based on the transformer architecture [41]. In contrast to traditional image segmentation models, which typically rely on CNNs, Segformer leverages the VIT as its core and harnesses its self-attention mechanism to achieve comprehensive understanding and focus on distinct regions within an image.

The process involves segmenting the image and converting the features of each segment into a sequence, which is then input into the transformer for further processing. At the heart of the transformer lies a Self-Attention Encoder comprising multiple Encoder layers, each housing a multi-head self-attention mechanism enabling nodes to aggregate attention weighted according to their individual features. The main core of the encoder is the self-attention layer. During the computation, the sequence is divided into multiple heads operating simultaneously. Each head *Q*, *K*, and *V* has the same dimensions *N* × *C*, where *N* = *H* × *W* is the length of the sequence and the estimation of the self-attention is

(1) $$Attention(Q,K,V)=Softmax(\frac{QK^{T}}{\sqrt{d_{head}}})V$$

The effect of zero-filling leakage of location information is considered in the model by means of a hybrid feed-forward network (Mix-FFN). The hybrid feed-forward network can be formulated as

(2) $$x_{out}=MLP\left(GELU\left({Conv}_{3\times 3}\left(MLP\left(x_{in}\right)\right)\right)\right)+x_{in}$$

where $x_{in}$ is the feature from the self-attention module.

This capability empowers the transformer to process both local and global information within the image. Through the stacking of multiple encoder layers, the transformer can extract multi-scale and multi-level features layer by layer, thus facilitating image segmentation. This approach not only effectively handles long-range dependencies within the image, but also captures global information more comprehensively, thereby enhancing the understanding of details and overall structure.

### 2.3. DLEF Module

**Figure 2:**
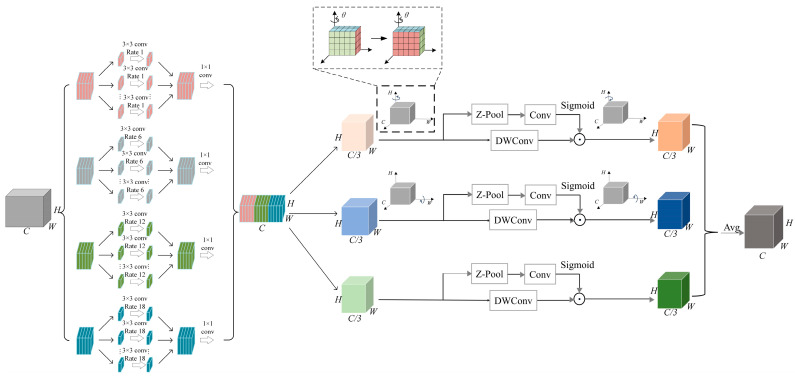
The structure of DLEF module.

As illustrated in Figure 2, the DLEF module initially partitions feature information into multi-scale features through dilated convolutions. These features subsequently undergo deep separation and reintegration to achieve structural reorganization of feature information, thereby enhancing their representational capacity. Finally, a group-wise rotation strategy based on the TA module [50] is implemented to establish cross-channel and spatial dimensional dependencies. Specifically, the strategy decouples the integrated information into three branches: the first two branches are rotated dimensionally along the width (*W*) and height (*H*) axes, respectively, while for the buildings of HRS images due to the disorder of the key feature points, the fixed angle of 90° is not able to be useful for the perfect construction of polymorphic spatial relationships, so we introduce the learnable parameter θ to apply different scene information. And the third branch retains the original spatial information to provide calibration processing for the rotated branch. Each branch is augmented by Z-Pool of residual connections to ensure a rich representation of the actual tensor. The method used in this paper is shifted from sequential rotation processing to parallel processing compared to the original TA module.

**Channel Attention Computation Branch**. The input features undergo Z-Pool, followed by a 7 × 7 convolution, and are then processed through a Sigmoid activation function to generate spatial attention weights.

**Channel *C* and Spatial *W* Dimension Interaction Capture Branch.** The input features are first permuted to transform into an H×C×W dimensional representation. Subsequently, Z-Pool is applied along the H dimension, followed by operations analogous to the first branch. Finally, the features are permuted back to a H×C×W dimensional format to facilitate element-wise addition.

**Channel *C* and Spatial *H* Dimension Interaction Capture Branch.** The input features are permuted to a W×H×C dimensional representation. Z-Pool is then applied along the W dimension, followed by similar operations as in the previous branches. The features are permuted back to a H×C×W dimensional format to enable element-wise addition. the Z-Pool is computed as

(3) $$Z-Pool(\chi )=[{MaxPool}_{d}(\chi ),{AvgPool}_{d}(\chi )]$$

where d is the dth dimension of the pooling operation.

### 2.4. MDC Module

**Figure 3:**
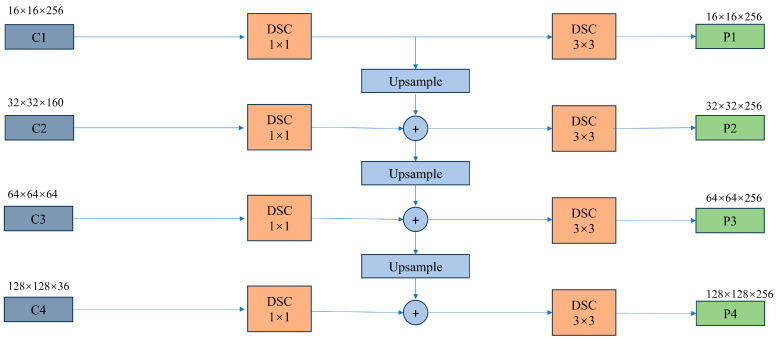
The structure of MDC module in the C_ASegformer.

In order to further integrate multi-scale semantic information, this study incorporates the MDC as the Neck layer. The MDC is responsible for Upsampling feature maps from lower scales and merging them with higher-scale feature maps, thereby generating a series of feature maps with diverse scales and resolutions [51]. Fundamentally, the MDC leverages information from various levels of the network’s feature maps and concurrently merges multi-scale feature maps to facilitate the detection of targets across multiple scales. The inclusion of the MDC layer significantly enhances the accuracy of target detection algorithms for multi-scale targets. Likewise, to circumvent overfitting in the model, this study replaces conventional convolution with DSC to minimize trainable parameters and mitigate overfitting concerns. The visual representation of these modifications is presented in Figure 3.

## 3. Experimental Datasets and Evaluation

To validate the accuracy of C_ASegformer in building extraction for clear and cloud-free conditions, we conducted experiments on three benchmark datasets: the Massachusetts Buildings Dataset, the INRIA Dataset, and the WHU Building Dataset, selected through comprehensive consideration of geographic diversity, sensor characteristics, and resolution adaptability. These datasets collectively span urban, suburban, and rural environments across North America, Europe, and Asia, ensuring geographic representativeness. Sensor diversity was addressed through the inclusion of satellite imagery (Massachusetts and INRIA) and aerial photography (WHU’s 0.3 m resolution UAV-collected data), covering ground resolutions from 0.3 m to 1 m to evaluate scale robustness.

### 3.1. Datasets

**Figure 4:**
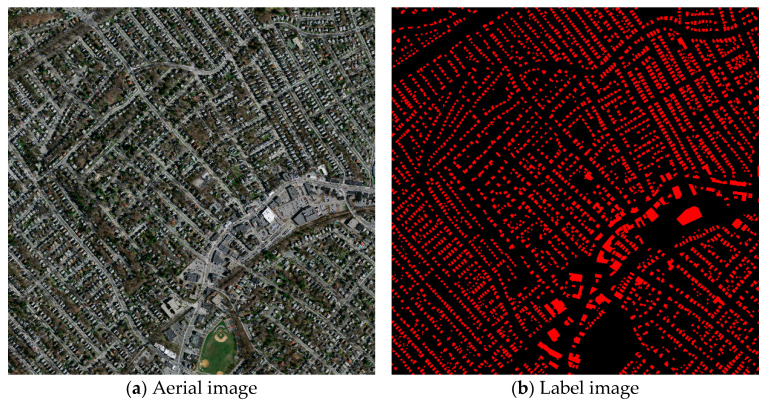
Examples of images and labels from the Massachusetts dataset.

The first dataset is the Massachusetts buildings dataset proposed by Mnih [47]. The Massachusetts buildings dataset consists of 151 aerial images of the Boston area, each covering roughly 2.25 square kilometers with a resolution of 1500*1500 and a spatial resolution of one meter. The 151 images in the dataset are divided into 137 training images, 4 validation images, and 10 test images. Each image consists of three channels (RGB) with original images and their corresponding labels as shown in Figure 4.

**Figure 5:**
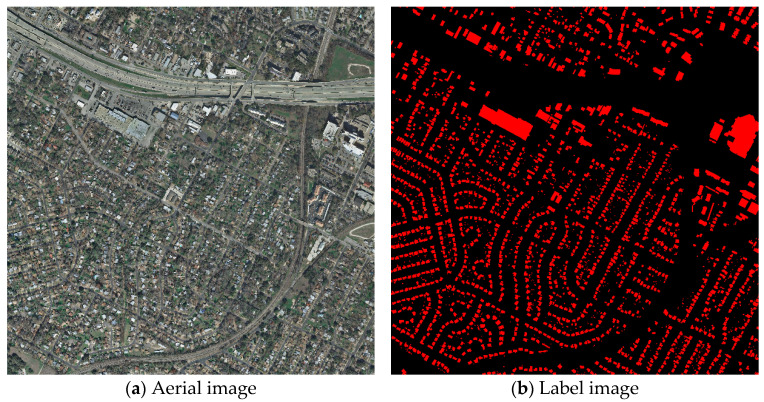
Examples of images and labels from the INRIA dataset.

The second dataset used is the INRIA dataset proposed by Maggiori [48], which covers building data from different cities in the world, with high resolution for each image. In this study, the dataset is divided into two parts: training and testing set, and the division ratio is 7:3. The aerial images and labels of the INRIA dataset are shown in Figure 5.

**Figure 6:**
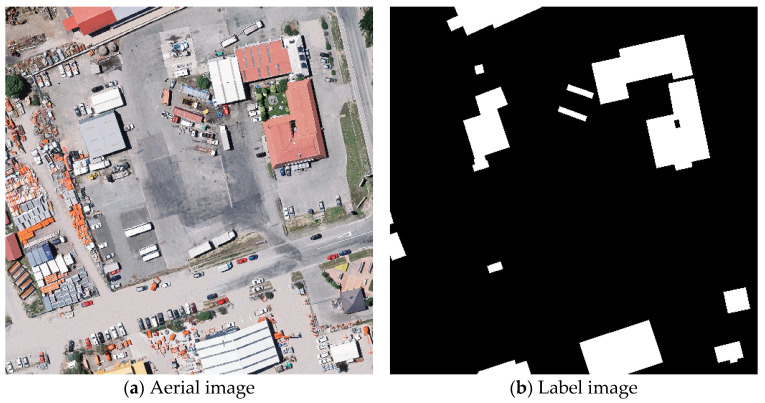
Examples of images and labels from the WHU dataset.

Finally, the WHU Building Dataset is a building dataset created by Wuhan University [49]. This dataset contains samples of buildings manually extracted from aerial and satellite imagery. The dataset is divided into training, validation, and test sets. Figure 6 shows the original images and their corresponding labels.

### 3.2. Data Processing

In order to enhance the model’s performance, this study necessitates an expanded dataset to enable the model to discern a broader spectrum of features for informed decision-making. Data augmentation, which generates new data from existing samples, is an effective strategy for improving model accuracy and mitigating overfitting.

Within the experiment, given the substantial resolution of the HRS image data, this study partitions each HRS image in the dataset into multiple 512*512 image segments. Subsequently, these images undergo random horizontal flipping with a 50% probability upon loading into the Dataloader. It is pivotal to note that the C_ASegformer model requires single-channel labels, while the dataset labels are represented as RGB three-channel images. Thus, in this study, each pixel’s channel gray value of 255 is assigned a value of 1, and any other gray value is assigned a value of 0. Consequently, within the dataset, pixels with a mask value of 1 signify the presence of buildings, whereas pixels with a mask value of 0 indicate background areas.

### 3.3. Experimental Settings

**Figure 7:**
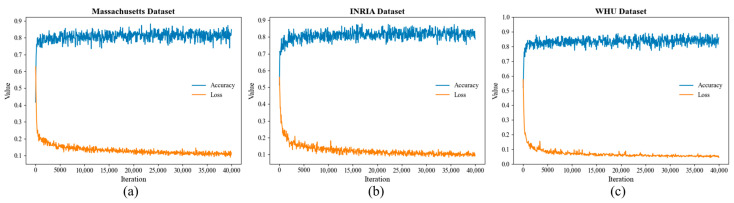
Accuracy and loss of models trained on each dataset: (**a**) Massachusetts dataset; (**b**) INRIA dataset; (**c**) WHU dataset. Accuracy curves are in blue, loss is in yellow curve.

All experiments in this paper were conducted on PyTorch’s framework (version 1.9.0). The experiment conducted multiple optimization experiments to ascertain the optimal hyperparameters. The specific settings of the hyperparameters are shown in Table 2. The training utilized the AdamW optimization function with an initial learning rate of 0.0006, and weight decay was incorporated to facilitate convergence and prevent overfitting. A total of 40,000 training iterations were experienced on the three dataset training sets and the training accuracy was verified on the validation set. To accommodate the GPU configuration, a batch size of 2 was set. Analysis of the three datasets training progress over 40,000 iterations revealed a gradual increase in overall accuracy coupled with a progressive decrease in loss. Additionally, localized fluctuations were observed within a small range. This trend is visually depicted in Figure 7.

### 3.4. Evaluation Metrics

This comparative trial assesses the accuracy of the models based on five evaluation criteria: ‘OA’, ‘Precision’, ‘Recall’, ‘F1-score’, and ‘IoU’ [52,53,54]. ‘IoU’ is typically computed for individual classes, and the ‘IoU’ of each class is accumulated and then averaged to provide a global evaluation [55]. The evaluation indicators used in this paper are expressed as

(4) $$Overall\;Accuracy=\frac{TP+TN}{TP+TN+FP+FN}$$

(5) $$Precision=\frac{TP}{TP+FP}$$

(6) $$Recall=\frac{TP}{TP+FN}$$

(7) $$F1=\frac{2\times Precision\times Recall}{Precision+Recall}$$

(8) $$IoU=\frac{TP}{TP+FP+FN}$$

where TP is true positive, TN is true negative, FP is false positive, and FN is false negative.

## 4. Results

In order to validate the effectiveness of the models, we selected several classical models (AGs-Unet, DeepLabV3+, MA-FCN, HRNet, MANet) as well as advanced Transformer-based models (Segformer, ST-UNet, CMTFNet, DSymfuser) for comparative experiments on the Massachusetts dataset, the INRIA dataset, and the WHU dataset for comparative experiments. All models used for comparative tests were trained and tested for evaluation in the same experimental environment.

### 4.1. Experimental Results on the Massachusetts Dataset

**Figure 8:**
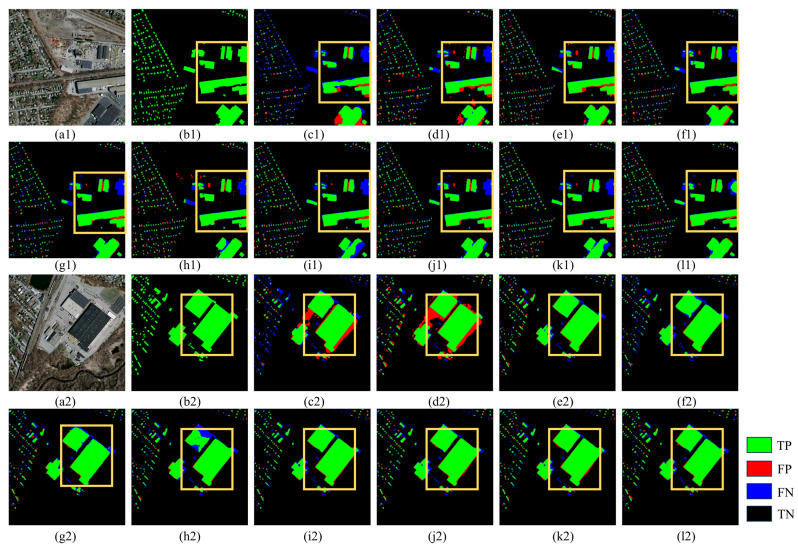
Visualization of segmentation performance on the Massachusetts dataset: (**a1–a2**) Input aerial images, (**b1–b2**) Ground truth labels, and (**c1–l2**) predictions from different deep learning models. Model abbreviations: (**c1**,**c2**) AGs-Unet, (**d1**,**d2**) DeepLabV3+, (**e1**,**e2**) MA-FCN, (**f1**,**f2**) HRNet, (**g1**,**g2**) Segformer, (**h1**,**h2**) MANet, (**i1**,**i2**) ST-UNet, (**j1**,**j2**) CMTFNet, (**k1**,**k2**) DSymFuser, (**l1**,**l2**) C_ASegformer. Yellow boxes indicate emphasis.

The Massachusetts dataset visualization is shown in Figure 8. C_ASegformer significantly outperforms the other models in terms of accuracy and completeness of building extraction. Compared to the misclassified regions as well as the missed regions appearing in AGs-Unet and MA-FCN, C_ASegformer’s correctly classified regions have more complete coverage and only have sporadic noise at the local edges, which indicates that it is more robust to complex building shapes and weakly textured regions. Compared to Transformer-based Segformer, C_ASegformer is more accurate in contour segmentation of small-sized buildings (e.g., detached garages) while avoiding the boundary sticking problem generated by HRNet and MANet in dense building clusters. It is worth noting that although DSymFuser and CMTFNet improve global consistency by capturing dependencies through Transformer, their red FPs are still more obvious in shaded areas (e.g., building projections), while C_ASegformer effectively suppresses such interference through top and bottom partitioning.

Table 3 demonstrates the model comparisons for the Massachusetts dataset, where C_ASegformer exhibits a significant overall performance advantage. In terms of key metrics, the model leads all compared models with 95.42% OA and 75.46% mIoU, which are 0.76% and 0.78% higher than the second-place DSymFuser, respectively, suggesting that it achieves the optimal global pixel classification and region segmentation accuracy. Notably, C_ASegformer has the highest recall (84.93%) of all models, which is 2.08% higher than MANet (82.85%), indicating its outstanding performance in reducing building omission detection. However, its precision (86.47%) is slightly lower than CMTFNet (87.64%) and DSymFuser (87.35%), implying that a small number of false detections may exist, but it still eventually outperforms the other models in terms of F1-score (85.69%) through the advantage of a high recall rate, reflecting a better balance of precision and recall.

### 4.2. Experimental Results on the INRIA Dataset

**Figure 9:**
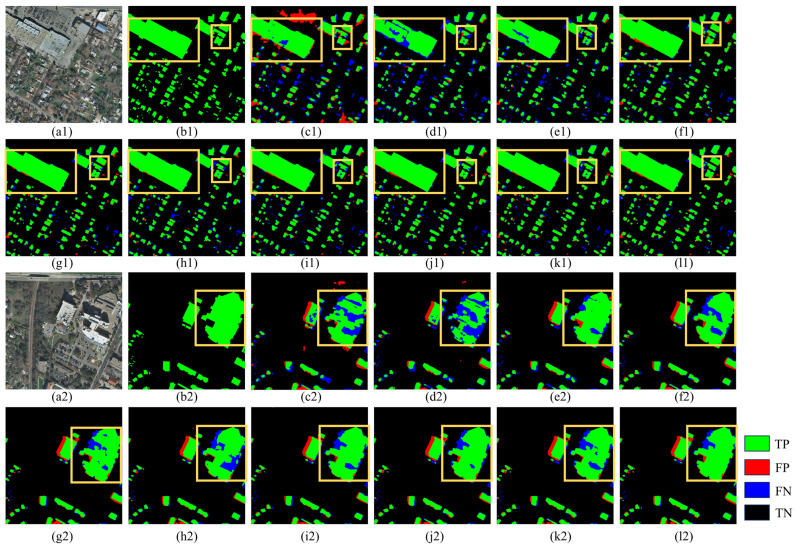
Visualization of segmentation performance on the INRIA dataset: (**a1**,**a2**) Input aerial images, (**b1**,**b2**) Ground truth labels, and (**c1**–**l2**) predictions from different deep learning models. Model abbreviations: (**c1**,**c2**) AGs-Unet, (**d1**,**d2**) DeepLabV3+, (**e1**,**e2**) MA-FCN, (**f1**,**f2**) HRNet, (**g1**,**g2**) Segformer, (**h1**,**h2**) MANet, (**i1**,**i2**) ST-UNet, (**j1**,**j2**) CMTFNet, (**k1**,**k2**) DSymFuser, (**l1**,**l2**) C_ASegformer. Yellow boxes indicate emphasis.

Figure 9 shows the performance of the comparison models on the INRIA dataset. C_ASegformer demonstrates significant robustness and accuracy advantages in building extraction tasks for complex urban scenes. Compared to other models, it has the most complete coverage of green TP regions, especially in low-contrast roofs and edge details of dense building clusters with almost no misdetection residuals, whereas AGs-Unet and MA-FCN have significant misdetections in such regions. In terms of misdetection control, C_ASegformer has the lowest number of misdetected areas and successfully avoids the problem of HRNet and Segformer misclassifying car park textures as buildings, as well as the sporadic noise produced by MANet in areas of dense vegetation. Notably, although DSymFuser and CMTFNet reduce large misclassified regions, they are still weaker than C_ASegformer in the continuity of fine buildings, which verifies the design effectiveness of its multi-scale feature synergy and context-aware module.

As shown in Table 4, in the comparison of building extraction models on the INRIA dataset, C_ASegformer tops the list with an mIoU of 82.58% and an F1-score of 89.97%, which is an improvement of 0.49% and 0.22% over the second-place DSymFuser (mIoU of 82.09% and F1 of 89.75%), respectively, indicating that it has the optimal region segmentation accuracy and comprehensive balancing ability. Although its precision (90.83%) is slightly lower than that of MANet (91.62%) and DSymFuser (91.44%), its recall of 89.17% is the highest of all models, with an improvement of 0.92% over that of DSymFuser (88.25%), which highlights its outstanding ability to reduce the omission of buildings in complex urban scenes. In addition, although C_ASegformer’s OA (95.33%) is only narrowly ahead of CMTFNet (95.24%), its significant improvement in mIoU (0.58% higher than CMTFNet) further validates its ability to capture the details of building boundaries.

### 4.3. Experimental Results on the WHU Dataset

**Figure 10:**
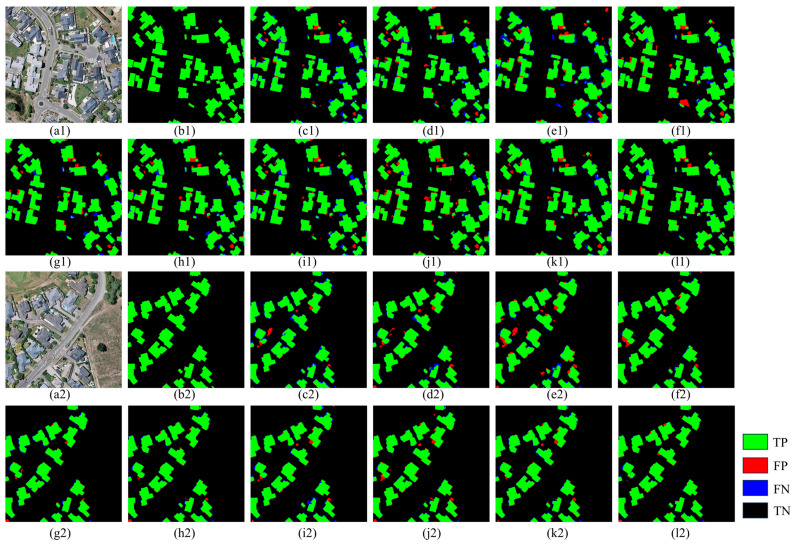
Visualization of segmentation performance on the WHU dataset: (**a1**,**a2**) Input aerial images, (**b1**,**b2**) Ground truth labels, and (**c1**–**l2**) predictions from different deep learning models. Model abbreviations: (**c1**,**c2**) AGs-Unet, (**d1**,**d2**) DeepLabV3+, (**e1**,**e2**) MA-FCN, (**f1**,**f2**) HRNet, (**g1**,**g2**) Segformer, (**h1**,**h2**) MANet, (**i1**,**i2**) ST-UNet, (**j1**,**j2**) CMTFNet, (**k1**,**k2**) DSymFuser, (**l1**,**l2**) C_ASegformer.

The visualization results for the WHU dataset are shown in Figure 10, where most of the models show good results. However, small buildings around large buildings in the comparison models are prone to information loss, and C_ASegformer effectively identifies small buildings through contextual relevance and long-distance dependency, proving the filtering ability of the models.

Table 5 shows the model comparison data for the WHU dataset, in which C_ASegformer demonstrates the overall leading performance. The model refreshes the list with an mIoU of 92.87% and an OA of 98.51%, which are 0.85% and 0.2% higher than the second-place CMTFNet (mIoU 92.02%, OA 98.31%) respectively, and especially achieves the highest F1-score (96.22%) and recall (96.18%), indicating that it segmented the high-density building regions and achieves the optimal balance of precision and completeness. Although MANet’s precision (96.44%) is slightly higher than that of C_ASegformer (96.26%), the latter eventually overtakes it by 0.35% on the F1-score through a significant advantage in recall (96.18%), highlighting its greater ability to capture complex building edges and small buildings. Notably, the mIoU of C_ASegformer reaches a peak of 92.87%, which is 0.85% and 0.94% higher than that of the Transformer-based CMTFNet (92.02%) and DSymFuser (91.93%), respectively, verifying the effectiveness of its improved attentional mechanism in multi-scale feature fusion.

## 5. Discussion

### 5.1. The Main Contribution of This Study

In this study, the comparative analysis between the mainstream model and C_ASegformer is conducted across three datasets, with C_ASegformer demonstrating exceptional performance in all instances. The segmentation results of the Massachusetts dataset are visually presented in Figure 8, highlighting the dataset’s densely distributed buildings and obscured building contours, which pose challenges for accurate segmentation. Similarly, the segmentation results of the INRIA dataset, depicted in Figure 9, demonstrate a looser yet irregular building distribution, further complicating the extraction process.

The observed segmentation results indicate that classical networks such as DeepLabV3+ exhibit reduced effectiveness in segmenting these challenging datasets. Additionally, the HRS image segmentation network MANet and the basic Segformer reveal shortcomings, including leakage within building boundaries and incomplete detection of occluded building edges.

Conversely, the C_ASegformer proposed in this study delivers promising results in both occluded edge segmentation and irregular building segmentation. Furthermore, the segmentation results of the WHU dataset, illustrated in Figure 10, showcase predominantly regular rectangular building shapes with a looser distribution, rendering the segmentation process comparatively simpler.

While both classical and emerging models demonstrate commendable performance, C_ASegformer excels in extracting detailed edge features of buildings and smaller structures. Thus, the C_ASegformer model proposed in this study represents significant progress in effectively addressing challenges related to the extraction of edge details and complex building structures across diverse datasets.

### 5.2. Ablation Study

In order to determine the efficacy of the DLEF module and the MDC module integrated into the Segformer model for building segmentation, this experiment was conducted on the Massachusetts dataset for ablation experiments. We conducted five experiments, and the means and variances obtained from the experiments are shown in Table 6, where, by focusing on local details after the introduction of DLEF, the OA, accuracy, and mIoU are improved by 0.26%, 0.39% and 0.49%, respectively. However, their lower recall rates indicate an increase in the leakage rate of complex targets, which may be due to the over-capturing of detailed information by DLEF. In contrast, when MDC acts alone, it improves global consistency by fusing multilevel features, but insufficient segmentation of small-scale buildings reduces the recall to 80.74%. However, when the two are enabled together, the inter-module effectively balances local details and global context to achieve a balanced model effect. Despite the slight reduction in OA, the recall increased significantly and the F1-score demonstrated the balancing ability of the model. Notably, mIoU, a key metric for evaluating the model, improved by 1.53% from baseline, reflecting the effectiveness of the modules in the building segmentation task.

**Figure 11:**
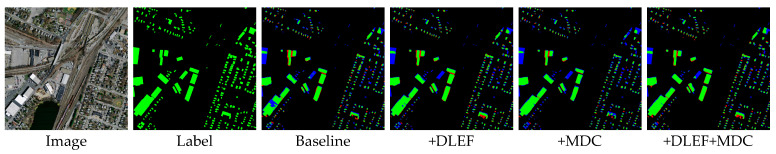
Visualization of ablation experiments on the Massachusetts dataset.

As shown in Figure 11, the DLEF module contributes to the segmentation of edge smoothness, while the MDC adeptly consolidates semantic information across multiple scales, effectively mitigating interior building leakage. Moreover, the combined utilization of both modules facilitates the fusion of diverse semantic information generated by attention mechanisms, thereby simultaneously addressing edge and interior aspects of building segmentation, ultimately culminating in improved accuracy.

### 5.3. Total Parameters of Different Networks

The aforementioned experiment undertakes a comprehensive comparison of the C_ASegformer model with other mainstream models across various dimensions, culminating in the discernment that the C_ASegformer model surpasses mainstream models in numerous facets, thereby significantly contributing to enhanced building segmentation accuracy. However, it is imperative to acknowledge that model parameters serve as pivotal indicators of model excellence. Notably, a lower parameter count corresponds to reduced time and memory space requirements during model training. Consequently, this experiment conducts a comparative analysis of the parameter count and computational load for each model to further elucidate the relationship between model parameters and accuracy.

**Figure 12:**
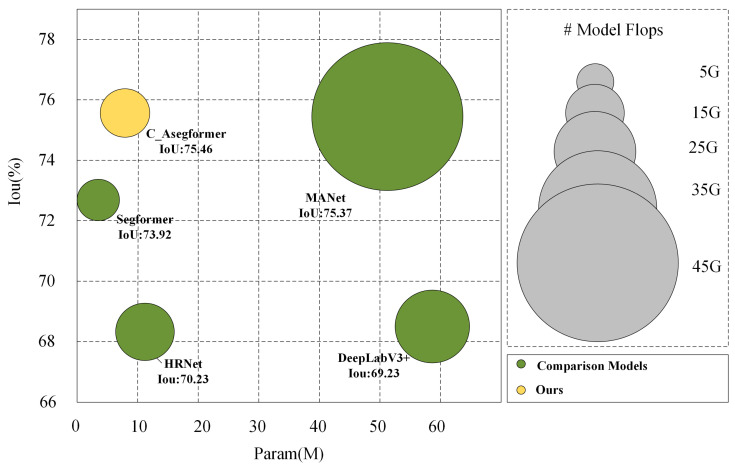
Complexity of each method on the Massachusetts dataset.

As shown in Table 7 and Figure 12, from the comprehensive comparison of the number of parameters, computational efficiency and inference speed, C_ASegformer shows significant advantages in lightweight semantic segmentation models. With only 5.56 M parameters and 11.47 G FLOPs, this model achieves a real-time inference speed of 38.6 f/s, and at the same time outperforms all the compared models with 75.46% mIoU, which reflects a breakthrough in the balance between accuracy and efficiency in the algorithm design. Compared to Segformer (3.71 M/7.79 G), which has a smaller number of parameters, C_ASegformer improves the mIoU by 1.54% at the cost of a small number of parameters through the structural optimization of parameter reduction, which verifies its effectiveness in improving the feature characterization capability without significantly increasing the computational burden. Notably, although MANet achieves a similar mIoU (75.37%), its computational cost of 48.21 M parameters and 44.35 G FLOPs results in an FPS of only 18.7, highlighting the usefulness of C_ASegformer in HRS scenarios.

### 5.4. Generalization Ability of C_ASegformer

In pursuit of a comprehensive exploration of the generalization capability of the C_ASegformer model, several networks underwent training on the Massachusetts building dataset’s training set. Subsequently, the trained networks were tested on a new test set comprising 200 randomly selected images from the INRIA dataset. As shown in Table 8, the Massachusetts dataset and the INRIA dataset contain different geographic areas and exhibit differences in spatial resolution, and therefore exhibit different architectural characteristics on the two datasets.

Table 9 delineates the test outcomes of the diverse networks. Notably, upon training on the Massachusetts dataset’s training set and subsequent testing on a fresh test set, the difference in generalization performance across datasets can be attributed to the significant difference in geostatistical characteristics between the Massachusetts and INRIA datasets: Massachusetts is dominated by low-density buildings in suburban areas of the US, while INRIA covers high-density urban areas in Europe, with a significant domain bias in spatial resolution, building scale distribution, and scene complexity. The CNN model (HRNet, DeepLabV3+) faces high-frequency detail loss in INRIA high-resolution data (IoU decreases by 37.36% and 36.01%, respectively) due to the limitations of local receptive fields and fixed-scale modelling, while the Transformer-based Segformer series effectively mitigates the feature mismatch and feature loss caused by the resolution difference by means of hierarchical positional coding and scale-invariant attention mechanism. Feature mismatch and building scale distribution bias due to resolution difference, among which C_ASegformer further introduces DLEF and shows better adaptation in INRIA dense building clusters, with IoU and F1-score improved by 1.84% and 5% compared to MANet, verifies the key role of global context modelling for cross-domain generalization.

### 5.5. Module Comparison

The model designed in this study utilizes a Transformer as the backbone to extract buildings from HRS images. It was observed that the multi-scale feature extracted by the Transformer is significantly affected by the complex environment of HRS images. To address this, we integrated a feature fusion module to merge the multi-scale feature information and mitigate the influence of interfering information. We employed the feature fusion module as the foundation and experimented with incorporating various attention mechanisms to better focus on the relevant information. Table 10 illustrates the impact of different feature fusion modules on accuracy. Among these, adding SE and CA did not demonstrate an improvement in accuracy. On the other hand, EA showed a notable contribution to the ‘OA’ index, although its performance in other indicators was mediocre. CBAM receives an effective boost by associating channels and spatial relations simultaneously; however, the TA module couples channels and spatial information more finely through rotational operations, making the effect even more pronounced.

## 6. Conclusions

This study proposed a novel Transformer-based semantic segmentation network model, termed C_ASegformer, which leverages Transformer as the backbone for image feature extraction. The model incorporates a DLEF module to effectively fuse global contextual information and fine-grained details from different receptive fields. Additionally, the TA module and MDC module are employed to enhance dependencies across various dimensions, thereby strengthening semantic contextual connections through comprehensive experiments on the Massachusetts, INRIA, and WHU datasets. On the Massachusetts dataset, our model achieved an ‘mIoU’ metric of 75.46%, outperforming other models. Specifically, it surpassed DeepLabV3+, HRNet, Segformer, MANet, ST-Unet, CMTFNet, and DSymfuser by 6.23%, 3.24%, 1.86%, 1.49%, 1.54%, 1.27%, and 0.78%, respectively. Additionally, the model maintained a moderate number of parameters and computational load. Compared to MANet, which has a similar accuracy but a parameter count of 48.21 M, C_ASegformer has a significantly lower parameter count of 5.56 M, highlighting its efficiency. The experimental results demonstrate that the model exhibits high accuracy and precision in extracting buildings from HRS images. It outperforms traditional convolutional neural network models in building segmentation tasks and effectively handles challenges posed by image noise.

Although our model performs well in various scenarios, it is still influenced by highly complex environmental conditions in HRS images. Additionally, the buildings themselves exhibit diverse shapes and sizes, further complicating the extraction process. Another limitation is the potential insufficiency of training datasets for certain specific HRS tasks. Transformer models in particular require a large amount of training data to perform effectively, which can lead to overfitting when training data are limited. In future work, we will focus on addressing the limitation of training data. We aim to explore the use of improved, smaller Transformer models as the backbone to enhance the model’s applicability across various scenarios.

## Figures and Tables

**Table 1:** Detailed architecture of each module of C_ASegformer, where ‘mlp_ratios’ represents the size of the hidden layer in the MLP; ‘sr_ratio’ controls the size of the *K*, *V* parameter matrix.

Framework	Module	Name	Number
Backbone	MiT-B0	patch_size	4
		embed_dims	[32, 64, 160, 256]
		num_heads	[1, 2, 5, 8]
		mlp_ratios	[4, 4, 4, 4]
		sr_ratios	[8, 4, 2, 1]
Neck	DLEF	dim_in	[32, 64, 160, 256]
		dim_out	[32, 64, 160, 256]
	MDC	in_channels	[32, 64, 160, 256]
		out_channels	256
		num_outs	4
Decoder_Head	Segformer_head	in_channels	[64, 128, 320, 512]
		in_index	[0, 1, 2, 3]
		feature_strides	[4, 8, 16, 32]

**Table 2:** Hyperparameter Setting.

Category	Parameter	Setting
Data Augmentation	Flip Probability	0.5 (Horizontal/Vertical)
	Rotation Range	±15°
	Crop Size	512 × 512
Optimizer (AdamW)	Initial Learning Rate	0.0006
	Beta Coefficients (β1/β2)	0.9/0.999
	Weight Decay	0.01
Training Protocol	Batch Size	2
	Training Iterations	40,000

**Table 3:** Quantitative comparisons with classical models on the Massachusetts dataset. The highest value for each metric is marked as bold.

	OA (%)	Precision (%)	Recall (%)	F1-Score (%)	mIoU (%)
AGs-UNet [56]	93.67	87.94	73.57	78.63	68.46
DeepLabV3+ [15]	93.34	84.73	79.1	81.82	69.23
MA-FCN [17]	94.12	87.07	82.89	84.93	73.8
HRNet [16]	93.98	85.82	82.01	83.87	72.22
Segformer [41]	94.36	86.93	82.38	84.59	73.60
MANet [18]	94.41	86.65	82.85	84.61	73.97
ST-UNet [44]	94.51	**87.68**	79.82	83.15	73.92
CMTFNet [45]	94.61	87.64	80.58	83.64	74.19
DSymFuser [46]	94.66	87.35	81.42	84.05	74.68
C_ASegformer	**95.42**	86.47	**84.93**	**85.69**	**75.46**

**Table 4:** Quantitative comparisons with different models on the INRIA dataset. The highest value for each metric is marked as bold.

	OA (%)	Precision (%)	Recall (%)	F1-Score (%)	mIoU (%)
AGs-Unet [56]	91.9	90.7	86.48	88.53	68.2
DeepLabV3+ [15]	93.28	87.35	86.4	86.88	76.8
MA-FCN [17]	92.48	88.25	86.11	87.17	77.14
HRNet [16]	94.12	90.33	87.53	88.91	80.03
Segformer [41]	95.21	90.67	88.77	89.69	81.16
MANet [18]	95.11	**91.62**	87.02	89.12	81.34
ST-UNet [44]	95.18	91.42	87.61	89.38	81.71
CMTFNet [45]	95.24	91.33	88.03	89.58	82.00
DSymFuser [46]	95.11	91.44	88.25	89.75	82.09
C_ASegformer	**95.33**	90.83	**89.17**	**89.97**	**82.58**

**Table 5:** Quantitative comparisons with classical models on the WHU dataset. The highest value for each metric is marked as bold.

	OA (%)	Precision (%)	Recall (%)	F1-Score (%)	mIoU (%)
AGs-Unet [56]	97.87	95.47	93.56	94.49	89.88
DeepLabV3+ [15]	98.25	95.56	95.58	95.57	91.73
MA-FCN [17]	96.8	94.8	94.4	94.2	89.1
HRNet [16]	98.15	95.67	94.89	95.27	91.21
Segformer [41]	98.31	95.78	95.66	95.72	91.99
MANet [18]	98.39	**96.44**	95.32	95.87	91.25
ST-UNet [44]	98.25	96.31	94.72	95.50	91.60
CMTFNet [45]	98.31	95.55	95.93	95.74	92.02
DSymFuser [46]	98.30	95.79	95.58	95.69	91.93
C_ASegformer	**98.51**	96.26	**96.18**	**96.22**	**92.87**

**Table 6:** Ablation experiments with modules which show the mean and variance of five experiments. √ means module added, × means module not added.

Model			OA (%)	Precision (%)	Recall (%)	F1-Score (%)	mIoU (%)
Baseline	DLEF	MDC					
√	×	×	94.34 ± 0.12	86.89 ± 0.35	82.21 ± 0.31	84.52 ± 0.28	73.86 ± 0.21
√	√	×	94.60 ± 0.10	87.28 ± 0.28	81.14 ± 0.33	83.78 ± 0.25	74.45 ± 0.19
√	×	√	94.54 ± 0.11	87.33 ± 0.30	80.74 ± 0.37	83.85 ± 0.31	74.19 ± 0.22
√	√	√	94.52 ± 0.15	84.74 ± 0.42	85.01 ± 0.25	84.83 ± 0.34	75.39 ± 0.18

**Table 7:** Comparison of parametric quantities and accuracy of each network.

	Params (M)	FLOPs (G)	FPS (f/s)	mIoU
MANet	48.21	44.35	18.7	75.37
Segformer	3.71	7.79	45.2	73.92
DeepLabV3+	58.6	23.99	12.5	69.23
HRNet	11.88	20.32	24.3	70.23
C_ASegformer	5.56	11.47	38.6	75.46

**Table 8:** Details of Massachusetts and INRIA datasets.

Statistical Indicators	Massachusetts	INRIA
Spatial resolution	1.0 m	0.3 m
Building density	Small	Large
Main building types	Stand-alone houses	Apartment complexes, commercial buildings
Percentage of vegetation cover	Large	Small

**Table 9:** Transfer learning results for each model on different datasets. The highest value for each metric is marked as bold.

Model	Massachusetts		INRIA	
	IoU (%)	F1-Score (%)	IoU (%)	F1-Score (%)
HRNet	70.23	81.96	32.87	50.25
DeepLabV3+	69.23	81.82	33.22	47.69
Segformer	73.92	84.59	65.13	74.94
MANet	75.37	84.61	64.26	75.24
C_ASegformer	**75.46**	**84.73**	**69.26**	**77.08**

**Table 10:** The accuracy of each module is compared using the Massachusetts dataset. The highest value for each metric is marked as bold.

Module	OA (%)	Precision (%)	Recall (%)	F1-Score (%)	mIoU (%)
Baseline	94.36	86.93	82.38	84.59	73.92
Baseline + DLEF + SE	94.55	**88.3**	79.34	83.05	73.49
Baseline + DLEF + CA	94.43	86.94	80.23	83.15	73.51
Baseline + DLEF + EA	94.66	87.2	81.67	84.14	74.79
Baseline + DLEF + CBAM	94.75	85.73	81.94	83.79	75.06
Baseline + DLEF + TA	**95.42**	86.47	**84.93**	**85.69**	**75.46**

## Data Availability

Data will be made available on request.
